# Exploring the impact of *Anaplasma phagocytophilum* on colonization resistance of *Ixodes scapularis* microbiota using network node manipulation

**DOI:** 10.1016/j.crpvbd.2024.100177

**Published:** 2024-04-28

**Authors:** Lianet Abuin-Denis, Elianne Piloto-Sardiñas, Apolline Maître, Alejandra Wu-Chuang, Lourdes Mateos-Hernández, Dasiel Obregon, Belkis Corona-González, Andréa Cristina Fogaça, Vaidas Palinauskas, Justė Aželytė, Alina Rodríguez-Mallon, Alejandro Cabezas-Cruz

**Affiliations:** a Animal Biotechnology Department, Center for Genetic Engineering and Biotechnology, Avenue 31 between 158 and 190, P.O. Box 6162, Havana, 10600, Cuba; b ANSES, INRAE, Ecole Nationale Vétérinaire d’Alfort, UMR BIPAR, Laboratoire de Santé Animale, Maisons-Alfort, F-94700, France; c Direction of Animal Health, National Center for Animal and Plant Health, Carretera de Tapaste y Autopista Nacional, Apartado Postal 10, San José de las Lajas, Mayabeque, 32700, Cuba; d INRAE, UR 0045 Laboratoire de Recherches sur le Développement de l'Elevage (SELMET-LRDE), 20250, Corte, France; e EA 7310, Laboratoire de Virologie, Université de Corse, Corte, France; f School of Environmental Sciences, University of Guelph, Guelph, ON, Canada; g Department of Parasitology, Institute of Biomedical Sciences, University of São Paulo, São Paulo, 05508-000, SP, Brazil; h Nature Research Centre, Akademijos 2, Vilnius, Lithuania

**Keywords:** Ticks, Tick-borne pathogens, Community assembly, Colonization resistance

## Abstract

Upon ingestion from an infected host, tick-borne pathogens (TBPs) have to overcome colonization resistance, a defense mechanism by which tick microbiota prevent microbial invasions. Previous studies have shown that the pathogen *Anaplasma phagocytophilum* alters the microbiota composition of the nymphs of *Ixodes scapularis*, but its impact on tick colonization resistance remains unclear. We analyzed tick microbiome genetic data using published Illumina 16S rRNA sequences, assessing microbial diversity within ticks (alpha diversity) through species richness, evenness, and phylogenetic diversity. We compared microbial communities in ticks with and without infection with *A. phagocytophilum* (beta diversity) using the Bray-Curtis index. We also built co-occurrence networks and used node manipulation to study the impact of *A. phagocytophilum* on microbial assembly and network robustness, crucial for colonization resistance. We examined network robustness by altering its connectivity, observing changes in the largest connected component (LCC) and the average path length (APL). Our findings revealed that infection with *A. phagocytophilum* does not significantly alter the overall microbial diversity in ticks. Despite a decrease in the number of nodes and connections within the microbial networks of infected ticks, certain core microbes remained consistently interconnected, suggesting a functional role. The network of infected ticks showed a heightened vulnerability to node removal, with smaller LCC and longer APL, indicating reduced resilience compared to the network of uninfected ticks. Interestingly, adding nodes to the network of infected ticks led to an increase in LCC and a decrease in APL, suggesting a recovery in network robustness, a trend not observed in networks of uninfected ticks. This improvement in network robustness upon node addition hints that infection with *A. phagocytophilum* might lower ticksʼ resistance to colonization, potentially facilitating further microbial invasions. We conclude that the compromised colonization resistance observed in tick microbiota following infection with *A. phagocytophilum* may facilitate co-infection in natural tick populations.

## Highlights

• *Anaplasma phagocytophilum* alters community assembly more than diversity in ticks.
• Disruption of community assembly was associated with reduced network robustness.
• Low network robustness links to weaker colonization resistance.
• Adding nodes improved network robustness in *A. phagocytophilum*-infected ticks.
• *A. phagocytophilum* reduces colonization resistance which may promote co-infections.

## Graphical abstract

**Figure:**
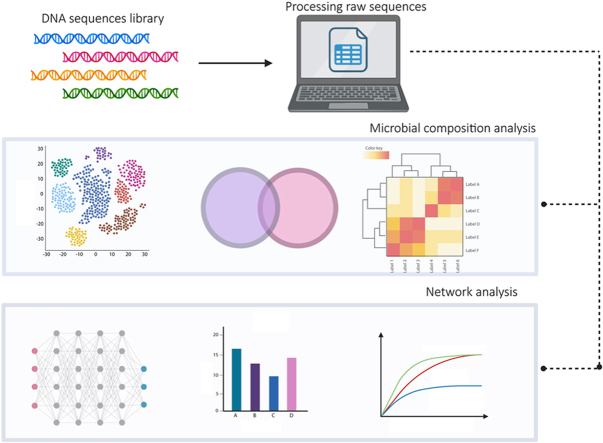

## 1 Introduction

Ticks are obligate hematophagous ectoparasites that transmit infectious agents, including bacteria (such as *Coxiella burnetti*, *Rickettsia helvetica*, *Borrelia burgdorferi*, and *Anaplasma marginale*), viruses (like severe fever with thrombocytopenia syndrome virus, tick-borne encephalitis virus, and Crimean-Congo haemorrhagic fever virus), and parasites (such as *Babesia microti* and *Theileria orientalis*) to terrestrial vertebrates (de la Fuente et al., 2017). Once acquired from an infected host, these tick-borne pathogens (TBPs) encounter several barriers, such as the peritrophic membrane, the dityrosine network, and tick immunity (Kurokawa et al., 2020; Fogaça et al., 2021; Kitsou et al., 2021). In addition to pathogens, ticks also carry non-pathogenic microorganisms (Bonnet et al., 2017), which include commensal microbes acquired from the environment, as well as transovarially-transmitted endosymbionts (Binetruy et al., 2020; Hussain et al., 2022), collectively referred to as microbiota (*sous rature*) (Cabezas-Cruz, 2023).

These non-pathogenic microorganisms gradually assemble over time, progressing from a state of low diversity to form richer multispecies communities that can have a substantial effect on the structure, organization, and function of the tick microbiota (Díaz-Sánchez et al., 2019; Obregón et al., 2019; Estrada-Peña et al., 2020a). In addition, non-pathogenic microorganisms may also play a role in driving the transmission of TBPs, which has significant implications for both human and animal health (Narasimhan et al., 2014; Gall et al., 2016; Wei et al., 2021; Wu-Chuang et al., 2021). There is empirical evidence for the presence of colonization resistance within the tick microbiota. Colonization resistance is the phenomenon where established microbial communities prevent the invasion and establishment of new, often pathogenic, species (Mullineaux-Sanders et al., 2018; Ducarmon et al., 2019; Stacy et al., 2021; Karita et al., 2022). For example, in *Dermacentor andersoni* ticks, the resident microbiota influences the acquisition levels of certain pathogens (Gall et al., 2016). Particularly, *Rickettsia bellii*, a non-pathogenic microorganism associated with *D. andersoni*, negatively correlates with *Anaplasma marginale* acquisition, indicating an antagonistic interaction (Gall et al., 2016). Additionally, experiments with *Haemaphysalis longicornis* ticks show that adult ticks emerging from nymphs treated with antibiotics exhibit a disrupted microbiota (Wei et al., 2021). These adult ticks had a higher infection rate of *Babesia microti* (44.7%) compared to control ticks (24.2%) (Wei et al., 2021), indicating that a healthy microbiota may play a crucial role in managing pathogen loads. This highlights that colonization resistance serves as a natural barrier against the establishment of TBPs in ticks.

Tick-transmitted bacterial pathogens likely evolved mechanisms to overcome colonization resistance by resident microbiota. For example, *Borrelia burgdorferi* colonization in *I. scapularis* increases the expression of several tick gut genes including *pixr*. Abrogation of PIXR function results in alterations in the gut microbiome, metabolome, and immune responses affecting the spirochete entering the tick gut (Narasimhan et al., 2014). Other mechanisms might involve triggering the expression of tick proteins with anti-microbial activity, such as *Ixodes scapularis* antifreeze glycoprotein (IAFGP), perturbing the tick gut microbiota (Abraham et al., 2017). This alteration affects the capacity of bacteria to form biofilms, influences the integrity of the peritrophic matrix, and reduces barriers to *Anaplasma phagocytophilum* colonization in the tick (Abraham et al., 2017). Overall, these mechanisms show an adaptation by TBPs to manipulate the tick microbial environment, effectively reducing colonization resistance and allowing for their proliferation and transmission. However, it remains unclear whether TBP-mediated modulation of tick microbiota results in community traits associated with reduced colonization resistance.

Altered microbial interactions and a weaker network structure have been proposed to lower colonization resistance (Shade et al., 2012). Disruptions in how these microbial communities are structured may reduce their overall stability and resilience, possibly affecting their capacity to fend off pathogens (Shade et al., 2012). A recent study by Maitre et al. (2022) found that an *R. helvetica* infection in the tick *Ixodes ricinus* significantly diminishes the diversity and connectivity within the tickʼs microbiota network, indicating a decrease in colonization resistance due to the infection Cohen and Havlin (2009).

In this study, we investigated colonization resistance of *I. scapularis* nymph microbiota infected with *A. phagocytophilum*. *Anaplasma phagocytophilum* is an obligate intracellular bacterium transmitted by ticks, causing human granulocytic anaplasmosis (Kocan et al., 2015). We hypothesized that alteration of the tick microbiota by *A. phagocytophilum* results in reduced colonization resistance, and diminished ability of the resident microbiota in the tick gut to prevent colonization by other microorganisms, including pathogens. To assess this hypothesis, we used published 16S rRNA amplicon sequencing data (Abraham et al., 2017) to compare the community assembly and robustness of *A. phagocytophilum-*infected and uninfected ticks, using a network approach. Co-occurrence networks (Estrada-Peña et al., 2020b; Mateos-Hernández et al., 2020, 2021, 2023; Maitre et al., 2023), with nodes representing individual microbial taxa and edges representing their interactions (Faust and Raes, 2012; Röttjers and Faust, 2018), were used to assess the impact of *A. phagocytophilum* on the tick microbial communities. Our findings indicate that infection with *A. phagocytophilum* reduced network robustness, potentially compromising colonization resistance. Interestingly, we observed a robustness recovery with the addition of new nodes in the *A. phagocytophilum-*infected network, suggesting that after infection, further microbial invasions may recover colonization resistance. These results are relevant for understanding the microbiota dynamics and responses to pathogen infections in ticks.

## 2 Materials and methods

### 2.1 Original datasets

**Fig. 1:**
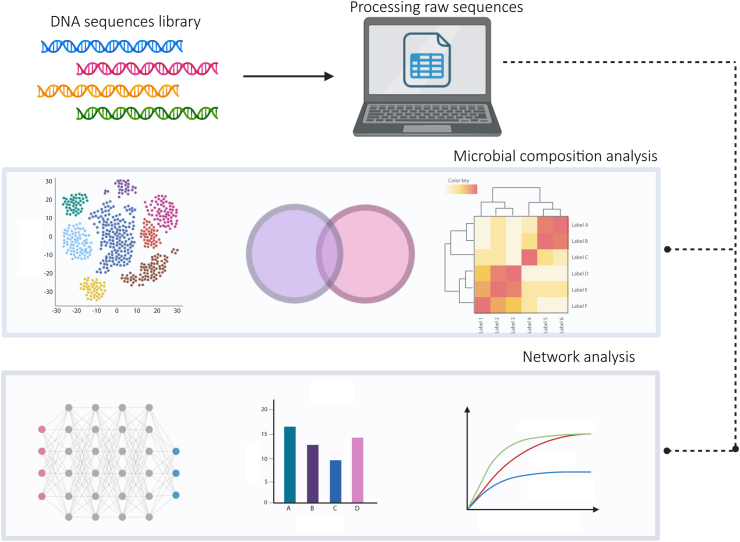
Experimental design. The data used in this study were downloaded from the SRA database and the raw sequences were processed. Bacterial composition and abundance were analyzed. The alpha- and beta diversity of the two datasets were compared, and co-occurrence networks were inferred to assess the structure of the microbial communities.

We evaluated the effect of bacterial infection on tick microbiota using publicly available datasets (Fig. 1). The selected studies utilized barcoded universal primers to amplify the V4 hypervariable regions of the 16S rRNA gene, followed by sequencing using the Illumina MiSeq system. The raw data were obtained from Abraham et al. (2017). In their study, the authors examined the changes in gut microbiota composition of *I. scapularis* nymphs fed on mice (C3H/SCID), which were experimentally infected with *A. phagocytophilum* (strain NCH-1). DNA was extracted from the guts of individual fed nymphs. The V4 variable region of the bacterial 16S rRNA was amplified from the genomic DNA of each sample using 12-base barcoded primer sets. In our study, we referred to *A. phagocytophilum* (Ap) tick groups as Ap-infected (*n* = 22) and Ap-uninfected (*n* = 10).

### 2.2 Processing of original raw sequences

We conducted taxonomic profiling of the 16S rRNA gene sequence datasets from the previously referenced studies. The sequences were sourced from the SRA repository: PRJNA353730. For sequence data processing, we used the pipelines in Quantitative Insights Into Microbial Ecology 2 Software (QIIME2) (version 2021.4.0) (Bolyen et al., 2019), as described in our previous publications (Estrada-Peña et al., 2020b; Wu-Chuang et al., 2023). In summary, the demultiplexed fastq files were denoised utilizing the DADA2 algorithm (version 1.18.0) as described by Callahan et al. (2016). Following this, all amplicon sequence variants (ASVs) were aligned employing the MAFFT tool (Katoh, 2002) through the q2-alignment interface. A phylogenetic tree was then generated using FastTree (Price et al., 2010) *via* the q2-phylogeny plugin. Diversity metrics, both phylogenetic and non-phylogenetic, were calculated based on a rarefied table of ASV abundances using the core-metrics-phylogenetic function within the q2-diversity plugin. Taxonomic annotation of the ASVs was carried out using the q2-feature-classifier's classify-sklearn naive Bayes taxonomy classifier, referencing the 16S rRNA SILVA database (release 132) (Quast et al., 2013) for alignment.

### 2.3 Diversity indices

To test for differences in bacterial diversity between Ap-uninfected and Ap-infected samples, we conducted analyses of alpha and beta diversity. For alpha diversity, we used the metrics “observed features” (DeSantis et al., 2006) and Faithʼs phylogenetic diversity index (Faith, 1992). Evenness was assessed using Pielouʼs evenness index (Pielou, 1966). The observed features metrics is the number of unique features present in a sample (DeSantis et al., 2006) and Faithʼs phylogenetic diversity index measures the cumulative evolutionary history represented in the community (Faith, 1992). Pielouʼs evenness index measures how evenly individuals are distributed among species within a community, indicating the balance of species abundances relative to species richness (Pielou, 1966). On the other hand, beta diversity measures the diversity between samples and examines the similarity in composition between analyzed communities. Beta diversity was evaluated using the principal coordinates analysis (PCoA), based on the Bray-Curtis dissimilarity index (Bray and Curtis, 1957). Unique and shared taxa among the conditions were visualized using Venn diagrams created with an online tool (http://bioinformatics.psb.ugent.be/webtools/Venn/) and the *Upset* package implemented in R v.4.3.1 (R Core Team, 2023). Analyses were performed using the RStudio Integrated Development Environment (IDE) v.2023.03.0-daily+82. pro2 (RStudio Team, 2020).

### 2.4 Inference of bacterial co-occurrence networks

Co-occurrence networks were employed to analyze bacterial interaction within microbial communities, both in Ap-uninfected and Ap-infected groups. These networks visually depict the relationships among interacting microbes, where an edge connects two bacteria if their abundances demonstrate significant correlation across samples under the same condition or treatment. The networks were constructed with taxonomic data tables at both family and genus levels (Supplementary file S2: Table S1), using the Sparse Correlations for Compositional data (SparCC) algorithm (Friedman and Alm, 2012), as implemented (Supplementary file S1) in the SpiecEasi R package (Kurtz et al., 2015). Only significant correlations between taxa, both negative and positive (SparCC, weight ≥ 0.5 or ≤ −0.5) were represented as edges. Furthermore, we also analyzed the strongest correlations (SparCC ≥ 0.9 or ≤ −0.9). Network metrics used in this study (Table 1) were computed using Gephi 0.9.5 (Bastian et al., 2009), an open-source software that transforms co-occurrence data in a graph.

**Table 1:** Key network metrics and their ecological interpretations in microbial community analysis.

Measure	Definition	Ecological interpretation
Network diameter	The network diameter measures the shortest path between the two most distant nodes in a network. It provides insight into the maximum number of edges required to connect any two nodes.	In microbial networks, a large diameter might suggest that some microbial interactions are spread over larger “distances”, implying less direct interaction between certain microbe taxa, potentially due to ecological or functional divergence.
Modularity	Modularity assesses the strength of network division into modules or communities. It quantifies the extent to which nodes in the same module are more densely connected to each other compared to nodes in different modules.	High modularity in a microbial network suggests that the network is divided into distinct communities or clusters, each possibly representing different ecological niches or functional groups within the microbiome.
Average degree	The average degree of a network determines the average number of edges per node. It reflects the overall connectivity of the network by measuring how many connections each node has on average.	In a microbial context, a higher average degree suggests a community where numerous microbe taxa interact with several others, which might imply a robust ecological network where many species are involved in maintaining community structure and function.
Weighted degree	The weighted degree takes into account the sum of edge weights connected to a node. It considers the strength or correlation intensity associated with each edge, providing a more nuanced view of node connectivity.	Considering the intensity or strength of connections, this metric provides insights into which microbe taxa play central roles based on the strength of their interactions.
Clustering coefficient	The clustering coefficient captures the tendency of nodes to form clusters or tightly interconnected groups. It measures the extent to which a nodeʼs neighbors are connected, indicating the presence of local clustering or community structures within the network.	A high clustering coefficient in microbial networks indicates a tendency of microbe taxa to form tightly knit groups, suggesting the presence of cooperative clusters or consortia.
Number of communities	Partitioning of nodes (representing entities or elements) within a network into distinct groups or communities based on their structural connectivity.	In microbial networks, each community might represent a different ecological niche or a group of microbe taxa performing similar functions.
Number of triangles	The number of triangles that exist within a given network. A triangle in a network is a set of three nodes that are all connected.	In microbial communities, triangles (three microbe taxa all interacting with each other) might suggest robust sub-communities that could stabilize the network against perturbations by providing redundant paths for interaction.
Largest Connected Component (LCC)	The LCC represents the main connected structure of the network. It identifies the largest subset of nodes that are mutually reachable through edges.	The size of the LCC in a microbial network indicates the core structure of connectivity and can reflect the main functional and structural backbone of the community.
Average Path Length (APL)	The average path length measures the efficiency of information flow within the network. It calculates the average number of steps required to travel between any two nodes in the network, indicating how quickly information can spread through the network.	In microbiological networks, the average path length relates to the efficiency of material or signal transfer across the network.
Degree centrality	Degree centrality measures the number of edges connected to a node, indicating the importance or influence of a node based on the number of connections it has.	In microbial networks, a high degree centrality indicates a species with numerous interactions, which could suggest a generalist species that engages with many different partners or a keystone species that plays a critical role in maintaining community structure and stability.
Betweenness centrality	Betweenness centrality quantifies the extent to which a node lies on the shortest paths between other nodes. It identifies nodes that act as intermediaries or bridges, facilitating communication in the network.	In ecological terms, species with high betweenness centrality may be those that link otherwise disparate groups of organisms, facilitating important ecological processes such as energy or material transfer across the community.
Closeness centrality	Closeness centrality measures how close a node is to all other nodes in the network. It reflects the efficiency of information flow from a node to other nodes, considering the shortest path lengths.	Species with high closeness centrality can quickly spread effects (either beneficial such as nutrients or detrimental such as pathogens) throughout the network, indicating their efficiency in influencing the community dynamics.
Eigenvector centrality	Eigenvector centrality considers both the local and global importance of a node. It assigns a centrality score to a node based on the centrality of its neighboring nodes, indicating its overall influence.	In a microbial community, such a metric would highlight species that are not only well-connected but also connected to other significant species, reinforcing their role in maintaining or disrupting complex community structures.
Hub taxa	Hub taxa are nodes in a network that exhibit high connectivity or act as hubs, having numerous connections with other nodes. They play a crucial role in maintaining network structure and information flow.	These taxa may represent species that provide essential ecosystem services, such as keystone species in ecological networks or core microbiota in host-associated microbial communities, whose loss might lead to drastic changes in network structure and function.
Core Association Network (CAN)	This network model identifies and visualizes the core set of interactions or associations between nodes (taxa) that are consistently present across multiple samples or conditions.	The CAN is useful for highlighting interactions that are critical to community structure and function, regardless of external conditions or perturbations.

### 2.5 Network comparisons

To compare networks, a statistical estimation analysis was conducted using Network Construction and Comparison with Microbes (*NetCoMi*) package (Peschel et al., 2021) in R v.4.3.1 (R Core Team, 2023) (Supplementary file S1), and performed using RStudio (RStudio Team, 2020). *NetCoMi* offers tools for networks alignment, which involves matching nodes (microbial taxa) and edges (co-occurrence relationships) between networks based on their topological properties helping to identify corresponding features between them, even if they are not identical. To assess similarities in the distribution of local centrality measures across nodes, i.e. degree-, betweenness-, closeness-, and eigenvector centrality (Table 1), between two networks, we computed the Jaccard index for each centrality measure. The Jaccard index measures the similarity between sets of “most central nodes”, i.e. nodes with a centrality value above the empirical 75th quartile, in the two networks. It expresses the similarity of the sets of most central nodes and the sets of hub taxa (highly connected nodes) between the two networks.

The Adjusted Rand Index (ARI) was also calculated to test the dissimilarity of clustering in the networks. Negative and positive ARI values range from −1 to 1, where values below 0 indicate lower than random clustering, values above 0 indicate higher than random clustering, a value of 1 corresponds to identical clustering, and a value of 0 indicates dissimilar clustering (Peschel et al., 2021).

Additionally, the Core Association Network (CAN) analysis (Röttjers et al., 2021), was performed using the Anuran software, implemented in Python environment (https://github.com/ramellose/anuran). The CAN analysis identifies conserved patterns across networks (Table 1), using a core prevalence threshold of 0.8. This approach utilizes null models to generate random networks and assesses the properties of these networks, allowing the identification of patterns in groups of networks. Our hypothesis was that CAN does not differentiate the microbial networks of Ap-uninfected and Ap-infected *I. scapularis* nymphs, indicating that infection does not lead to altered core associations in the microbial network. CAN visualization was carried out using Gephi 0.9.5 (Bastian et al., 2009).

### 2.6 Network robustness analysis

We evaluated the robustness of microbial co-occurrence networks to perturbation by measuring the impact of removing or adding nodes on network connectivity. To assess this, we simulated the proportion of node removal required to reach a loss in connectivity of 0.80 for each network using random or directed attacks. For the directed attack, we employed three strategies: betweenness centrality, degree centrality, and cascading. In the betweenness centrality approach, we removed nodes with the highest betweenness centrality values. In the degree centrality approach, we removed nodes with the highest degree centrality values. In the cascading approach, we first removed nodes with the highest betweenness centrality values, recalculating betweenness centrality after each node removal. To perform the network robustness analysis, we utilized the Network Strengths and Weaknesses Analysis (*NetSwan*) package (Lhomme, 2015) in R v.4.3.1 (R Core Team, 2023) (Supplementary file S1), performed using the RStudio (RStudio Team, 2020).

Additionally, we conducted a node addition analysis based on the method described by Freitas et al. (2020) in R v.4.3.1 (R Core Team, 2023) (Supplementary file S1), and performed using the RStudio (RStudio Team, 2020). In this analysis, new nodes were randomly selected and connected to the existing network. We then calculated the size of the largest connected component (LCC) and the average path length (APL) (Table 1). To obtain a more accurate estimate of the networkʼs robustness, we repeated the simulation with different sets of nodes, adding 100, 300, 500, 700, and 1000 nodes. The obtained values were plotted using GraphPad Prism 8.0.1 to visualize the results.

### 2.7 Statistical analysis

Differences in alpha diversity between groups were assessed using the Kruskal-Wallis test (*P* < 0.05) in QIIME2 (Bolyen et al., 2019). The Bray-Curtis dissimilarity index values were compared between groups using a PERMANOVA test (*P* < 0.05). A PERMANOVA test (*P* < 0.05) was performed to analyze beta dispersion. Additionally, we calculated beta dispersion (variance) using the betadisper function from the *Vegan* package in R v.4.3.1 (R Core Team, 2023) (Supplementary file S1), and performed using RStudio (RStudio Team, 2020).

The differences in bacterial taxa abundance between the Ap-uninfected and Ap-infected groups were performed with the ANOVA-Like Differential Expression (ALDEx2) method (Fernandes et al., 2013) implemented in R v.4.3.1 (R Core Team, 2023), and performed using RStudio (RStudio Team, 2020). Relative abundance was measured as centered log-ratio (clr) transformation which uses the geometric mean of the read counts in the sample. The advantage of the clr-transformation is that it makes the quantification scale-free and therefore comparable between conditions (Fernandes et al., 2013). The resulting data were used to construct the heatmap with the heatmap.2 function (Supplementary file S1), implemented in R v.4.3.1 (R Core Team, 2023), and performed using RStudio (RStudio Team, 2020). The comparisons were performed with Welchʼs *t*-test (*P* ≤ 0.05).

To test the similarity in the distribution of local centrality measures between two networks, two *P*-values, namely *P* (J ≤ j) and *P* (J ≥ j), were computed for each local centrality measure (Real and Vargas, 1996). These *P*-values represent the probability that the observed Jaccard index (J) value is either “less than or equal to” or “greater than or equal to” the expected Jaccard value at random (j). Differences were considered significant when *P* < 0.05.

The standard error for loss of connectivity was calculated, considering variability, using a threshold of 0.975. Additionally, the node addition analysis employed Wilcoxon signed-rank tests to determine if the mean size of the LCC and the APL differed significantly from 0. The *P*-values from these tests were adjusted using the Benjamini-Hochberg (BH) procedure to control for multiple comparisons. Additionally, bootstrapping was performed to obtain confidence intervals for the variables. Significance was determined at a threshold of *P* < 0.05.

## 3 Results

### 3.1 Impact of *A. phagocytophilum* on bacterial diversity and composition of tick microbiota

**Fig. 2:**
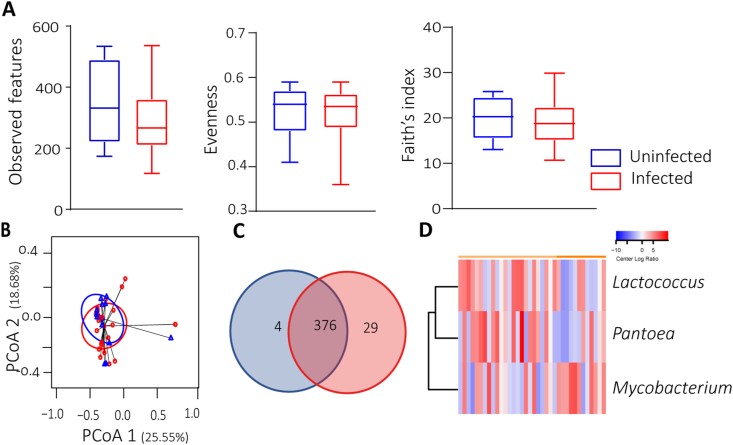
Impact of *A. phagocytophilum* infection on microbiome diversity. **A** Comparison of the alpha diversity by observed features (Kruskal-Wallis test, *P* = 0.31), Pielouʼs evenness index (Kruskal-Wallis test, *P* = 0.90) and Faithʼs index (Kruskal-Wallis test, *P* = 0.46). **B** Comparison of beta diversity for Ap-uninfected *vs* Ap-infected using the Bray-Curtis dissimilarity index (PERMANOVA, *F* = 32.84, *P* = 0.087, stress = 0.1507). Small circles and triangles in the principal coordinate analysis (PCoA) plot represent samples; ellipses indicate 95% confidence intervals. ANOVA test was performed and showed that the beta dispersion of the samples is not significantly different (*P* = 0.849). **C** Venn diagram showing the number of unique or shared taxa between Ap-uninfected and Ap-infected ticks. **D** Dendrogram heatmap resulting from the heatmap.2 functions implemented in R (R Core Team, 2023), and performed in RStudio IDE (RStudio Team, 2020). The taxa were clustered based on relative abundance (calculated as clr-transformed values). Each column represents the clr-values for bacterial taxa per sample and group. Lines represent bacterial taxa with significant changes between the two datasets. Color represents the clr-value.

The results indicated that there were no significant differences in alpha diversity (Fig. 2A) and beta diversity (Fig. 2B) between the Ap-uninfected and Ap-infected conditions (Kruskal-Wallis, *P* > 0.05; PERMANOVA, *P* > 0.05). In terms of microbiota composition, a total of 409 bacterial taxa were identified (Supplementary file S2: Table S2). Of these, 4 bacterial taxa (0.98%) were exclusive to the Ap-uninfected group, 29 bacterial taxa (7.10%) were exclusive to the Ap-infected group, and 376 bacterial taxa (91.9%) were shared between the two groups (Fig. 2C). Next, we conducted a differential abundance analysis to identify changes in specific taxa between Ap-uninfected and Ap-infected groups. We observed a significantly different abundance of three taxa, *Lactoccus*, *Pantoea* and *Mycobacterium,* across the two conditions (Fig. 2D).

### 3.2 Impact of *A. phagocytophilum* on bacterial community assembly

**Fig. 3:**
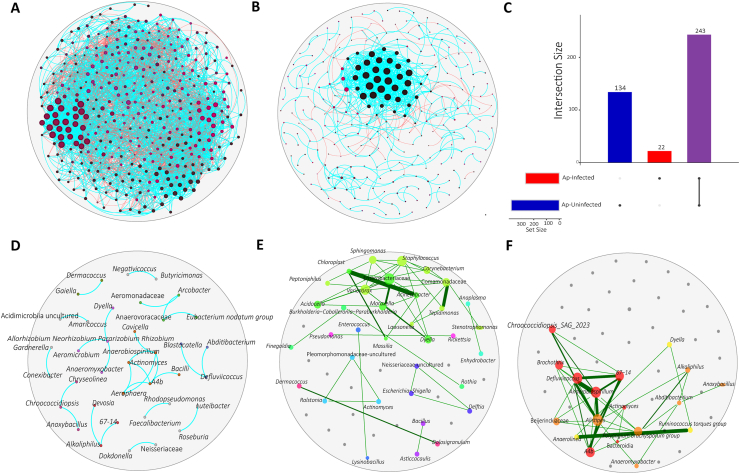
Networks representing community assemblies. Co-occurrences networks of Ap-uninfected (**A**) and Ap-infected (**B**). Nodes correspond to taxa (family or genus level), and connecting edges indicate a correlation between them. Only nodes with at least one significant correlation are represented. Node colors are based on modularity class metric and equal color means modules of co-occurring taxa. Node sizes are based on eigenvector centrality. Edges represent positive (*blue*) or negative (*red*) correlations (SparCC ≥ 0.5 or ≤ −0.5). **C** Number of unique or shared taxa in the Ap-uninfected and Ap-infected network. D Core Association Network (CAN) between Ap-uninfected and Ap-infected groups. **E**, **F** Strong correlation networks (weight > 0.9 or < −0.9) across the Ap-uninfected (**E**) and Ap-infected networks (**F**). Nodes correspond to taxa (family or genus level) and connecting edges indicate significant correlation between them. Edges represent positive (*green*) correlations; edge widths are proportional to correlation coefficients.

We evaluated the impact of *A. phagocytophilum* infection on tick bacterial communities using co-occurrence networks. Visual examination of the networks (SparCC, weight ≥ 0.5 or ≤ −0.5) of Ap-uninfected (Fig. 3A) and Ap-infected (Fig. 3B) revealed topological differences. Out of 409 bacterial taxa that were found in the microbiota, there were 377 (92.2%) nodes in the Ap-uninfected network and 265 (64.8%) nodes were present in the Ap-infected network (Table 2). Analysis of the topological features of the networks revealed a higher number of nodes and edges in the Ap-uninfected network (Fig. 3A, Table 2) than in the Ap-infected network (Fig. 3B, Table 2). Ap-infected network presented a reduction of 30% (265 out of 377) of the number of nodes and 82% (826 out of 4605) of the number of edges compared with Ap-uninfected network (Table 2). Furthermore, in both network conditions, positive interactions between the nodes were predominant (Table 2). Upon infection, the microbial assembly underwent reorganization, resulting in a decrease in the modularity and an increase in the number of distinct communities (Table 2). There were 243 taxa (61%, total 399) shared between the two networks, while 134 (33.6%) and 22 (5.4%) were unique to the Ap-uninfected and Ap-infected networks, respectively (Fig. 3C, Table S3).

**Table 2:** Topological features of the taxonomic networks of Ap-uninfected and Ap-infected groups.

Network features	*Anaplasma phagocytophilum*	
	Ap-uninfected	Ap-infected
No. of nodes	377	265
No. of edges	4605	826
Positive interactions	2844 (61.75%)	764 (92.49%)
Negative interactions	1762 (38.26%)	62 (7.50%)
Modularity	1.753	0.4
Number of communities	6	62
Network diameter	6	18
Average degree	24.43	4.089
Weighted degree	4.038	2.305
Clustering coefficient (Triangles method)	0.436	0.699
No. of triangles	20,375	3910
Connectivity	1	24

The Jaccard index was used to evaluate the similarities in selected local network centrality measures between Ap-uninfected and Ap-infected networks. The results showed that the Jaccard index of all local centrality measures was significantly lower (*P* ≤ Jacc) than expected by random for the comparisons of Ap-uninfected *vs* Ap-infected networks (Table 3). The ARI similarity index was employed to assess clustering differences between networks. The ARI supported the low similarity in clustering between Ap-infected and Ap-uninfected networks (ARI = −0.001, *P* = 0.891). The Core Association Network (CAN) revealed 40 core-interacting nodes (Fig. 3D, Supplementary file S2: Table S4), which represents a reduction of 89.4% and 85.0% in the number of nodes in Ap-uninfected and Ap-infected networks, respectively. This supported that infection causes a major impact in the tick microbial network, but also that a core set of interactions critical to community structure and function remains regardless of perturbation. Among these core-associated nodes, only positive interactions were found.

**Table 3:** Jaccard index for infected and uninfected tick networks.

Local centrality measures	Jaccard index and statistical significance of differences		
	Jacc	*P* (≤ Jacc)	*P* (≥ Jacc)
Degree centrality	0.12	0.001***	1.00
Betweenness centrality	0.14	0.001***	1.00
Closeness centrality	0.14	0.001***	1.00
Eigenvector centrality	0.14	0.001***	1.00
Hub taxa	0.32	0.46	0.58

*Notes*: *P* (≤ Jacc) and *P* (≥ Jacc) refer to the probabilities of observed Jaccard similarity coefficient (Jacc) that is either less than or equal to, or greater than or equal to, a Jacc calculated for randomly generated networks, respectively. Significance code: ***P = 0.001.

Finally, we examined the presence of strong correlations between network nodes. In the Ap-uninfected network, we identified 34 nodes with the strongest connections (Fig. 3E), and in the Ap-infected network 18 nodes were identified as the strongest connected (Fig. 3F). In the Ap-infected network, the nodes formed a major cluster, but this pattern was not observed in the Ap-uninfected network. Overall, these findings suggest significant alterations in the assembly of tick bacterial communities due to the presence of pathogens, which is supported by the observed topological differences, dissimilarity in local network centrality measures, and network clustering.

### 3.3 Impact of *A. phagocytophilum* on the network robustness

**Fig. 4:**
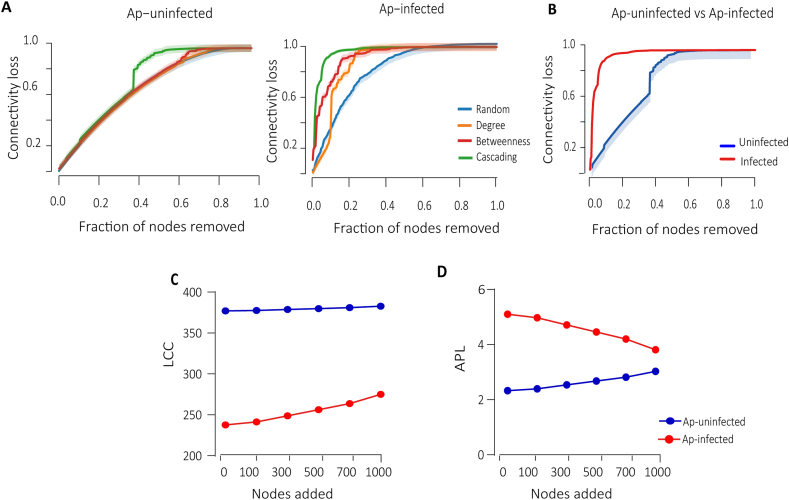
Comparison of network robustness after node addition and removal. **A** Connectivity loss was measured in different attack scenarios for Ap-uninfected and Ap-infected networks: betweenness (*red*), cascading (*green*), degree (*orange*), and random (*blue*). **B** Cascading removal representation for Ap-uninfected *vs* Ap-infected. Additionally, the impact of node addition on network robustness was evaluated by employing two measures largest connected component (LCC) and average path length (APL) (**C**, **D**). For each network, a total of 1000 nodes were added. The value reached for LCC size (**C**) and the APL (**D**) for *A. phagocytophilum* after each node addition was plotted. Tick groups (uninfected and infected) are represented by blue and red, respectively.

One crucial aspect of networks is their ability to withstand perturbations, including the removal or addition of nodes. In terms of connectivity loss, the removal of nodes through direct attack had a more significant impact on all networks compared to random attacks (Fig. 4A). Cascading removal had the most profound effect on network connectivity (Fig. 4B). In the Ap-infected network, 0.05 fraction of nodes removed was enough to achieve a connectivity loss of 0.80 (Fig. 4B). In contrast, the Ap-uninfected network required the removal of a larger fraction, specifically 0.38 of nodes, to reach the same level of connectivity loss (Fig. 4B). The results also showed that the networks of uninfected ticks maintain a larger largest connected component (LCC) (Fig. 4C) and have shorter average path length (APL) on average (Fig. 4D) compared to the infected ones. This suggests that the infection reduces network robustness.

However, after adding nodes, the Ap-infected network showed a notable improvement in the LCC (Fig. 4C) and a reduction in the APL (Fig. 4D), indicating a recovery of robustness. In contrast, the Ap-uninfected network showed an increase in the average path length after adding nodes (Fig. 4D), which implies a slight decrease in robustness. Overall, these findings point out the vulnerabilities in microbial communities of ticks infected with *A. phagocytophilum* as well as their ability to regain robustness after eventual microbial invasions.

## 4 Discussion

The colonization resistance of tick microbiota may play a pivotal role in shaping the dynamics of tick-borne diseases. In this study, we propose that infection with *A. phagocytophilum* significantly restructures the tick microbial community, potentially leading to reduced colonization resistance. To explore this hypothesis, we employed a network-based approach, with a specific focus on network robustness, to assess the impact of *A. phagocytophilum* on colonization resistance.

We observed no significant differences in alpha diversity metrics, including richness, evenness, and phylogenetic diversity, or in beta diversity using the Bray-Curtis dissimilarity index between the Ap-uninfected and Ap-infected groups. However, in contrast to our findings, Abraham et al. (2017) observed beta diversity differences between the Ap-uninfected and Ap-infected groups using the weighted UniFrac metric. This difference underscores the impact of employing different analytical pipelines in the analysis of microbial community data. For instance, while we employed amplicon sequence variants (ASVs), Abraham et al. (2017) used operational taxonomic units (OTUs). ASVs capture finer-scale diversity due to their resolution at the sequence level, whereas OTUs may lump together closely related sequences into the same unit, potentially obscuring underlying diversity patterns (Chiarello et al., 2022). This choice of pipeline can also influence alpha- and beta diversities, altering the ecological signal detected, particularly regarding presence/absence indices like richness index and unweighted UniFrac (Chiarello et al., 2022). This discrepancy in diversity estimates may be associated with the lower evenness of relative abundances and a higher number of rare units observed with OTUs compared to ASVs (Chiarello et al., 2022). Furthermore, the correlation between microbial communities varied across different beta diversity indices (Nearing et al., 2018; Chiarello et al., 2022). For instance, weighted UniFrac emphasizes phylogenetic relationships between microbial taxa, revealing patterns of community assembly driven by evolutionary processes. This metric is particularly useful for understanding how closely related taxa are distributed across different samples (Parks and Beiko, 2013). On the other hand, Bray-Curtis focuses on the abundance and presence of taxa, providing insights into compositional differences between samples (Ricotta and Podani, 2017). It is valuable for identifying which taxa contribute most to dissimilarities between communities and can highlight ecological factors shaping community composition. The contrasting results obtained using Bray-Curtis dissimilarity and weighted UniFrac metrics highlight the substantial impact of employing different analytical pipelines in microbial community analysis. The choice between ASVs and OTUs as well as the selection of diversity indices can significantly influence the ecological signals detected, particularly in relation to presence/absence metrics and phylogenetic relationships.

The lack of significant changes in alpha diversity suggests that overall microbial richness and evenness may remain relatively stable despite the presence of *A. phagocythophilum*. However, the observed change in composition (Fig. 2C) after the infection suggests that *A. phagocythophilum* could affect the occurrence of specific taxa in the tick microbiota, which may have important implications for colonization resistance. Colonization resistance is not dependent on a single species but rather on the associations of multiple bacteria cohesively living in a community (Spragge et al., 2023). These findings suggest that while the overall diversity of the microbiota may not be impacted, specific microbial communities crucial for colonization resistance could be compromised by infection with *A. phagocythophilum*, as evidenced by the present network analysis.

The observed composition changes could be associated with immunomodulation mediated by *A. phagocytophilum* infection (Abraham et al., 2017). *Ixodes scapularis* antifreeze glycoprotein (IAFGP), a protein induced by *A. phagocytophilum* in ticks, selectively binds the terminal d-alanine residue of Gram-positive bacteria and inhibits biofilm formation among Gram-positive pathogens like *Staphylococcus aureus*, correlating with a reduction in Gram-positive biofilm-forming species, including enterococci, during *A. phagocytophilum* infection (Abraham et al., 2017). The differential role of IAFGP on Gram-positive *versus* Gram-negative bacteria provides additional insights into the observed effects of IAFGP on the tick microbiota and specific genera, particularly in the context of *A. phagocytophilum* infection in ticks. Notably, silencing *iafgp* impaired *A. phagocytophilum* colonization of the tick gut (Abraham et al., 2017), suggesting that reduced colonization resistance mediated by IAFGP is essential for pathogen infection. Despite the stability in the overall number of observed features, the altered microbial composition may compromise functional redundancy (Estrada-Peña et al., 2020a), potentially weakening the tick microbiotaʼs ability to resist colonization by pathogens.

As previously reported for other intracellular pathogens (Maitre et al., 2022, 2023), infection reduces network complexity, which agrees with our findings of *A. phagocytophilum* infection being linked to a reduction in the number of nodes and edges in the microbial networks. The varying proportions of unique and shared nodes between the Ap-uninfected and Ap-infected networks suggest that infection changes the composition of the bacterial community resulting in changes in network structure, and interactions among bacterial species within the community assembly. Nevertheless, the presence of shared nodes between the Ap-uninfected and Ap-infected networks, indicates that certain microbial taxa persist and maintain their interactions despite the presence of pathogens. These shared taxa may play important roles in maintaining the stability of the community (Seal et al., 2021; Paulino et al., 2023).

The observation that only positive interactions remained in the core network suggests a cooperative relationship among the microbial taxa (Chow et al., 2014; Fountain-Jones et al., 2023). Positive interactions can indicate functional associations where organisms perform similar or complementary functions or interactions shaped by interspecies cross-feeding (Fuhrman and Steele, 2008; Eiler et al., 2012; Chow et al., 2014; Lejal et al., 2021). Such cooperative interactions are thought to contribute to the overall stability and functionality of the microbial community, potentially enhancing colonization resistance (He et al., 2014; Aivelo et al., 2019; Oña and Kost, 2022). Negative interactions can reflect competition and niche partitioning among microorganisms (Engel and Moran, 2013). While negative interactions may disrupt the stability of the network to some extent, their presence suggests ongoing ecological dynamics within the community that could benefit the tick. For example, antagonistic interactions between gut microorganisms in insects can potentially have protective functions against pathogens (Engel and Moran, 2013).

Microbial communities, like those within tick populations, play a crucial role in the ecosystem balance, affecting both disease transmission and resistance (Wu-Chuang et al., 2021, 2023; Pavanelo et al., 2023). The stability of microbial networks in the face of disturbances - such as infections - can significantly influence their function and resilience (Shade et al., 2012; Estrada-Peña et al., 2020b). In our study, using network analysis and principles of percolation theory, we assess the microbial networksʼ resistance to various types of disruptions, including node removals (Cohen et al., 2000, 2001) and additions (Cohen and Havlin, 2009), and how these disruptions affect network connectivity and efficiency. Network robustness reflects a systemʼs ability to maintain its connectivity and function despite disturbances (Cohen et al., 2000, 2001; Cohen and Havlin, 2009). Our findings indicate that infection with *A. phagocytophilum* compromises the network robustness, demonstrated by a more significant loss of connectivity following node removals. This suggests that the infection makes the microbial community more vulnerable to disturbances, with a potential decrease in colonization resistance.

On the other hand, after the introduction of new nodes (simulating the addition of new microbial species), we observed that infected tick networks exhibited lower LCC and higher APL values. The LCC is a measure of network cohesiveness, representing the largest group of interconnected nodes (Barabási and Pósfai, 2016; Kitsak et al., 2018), whereas APL indicates the networkʼs efficiency, with shorter paths meaning quicker and more efficient communication between nodes (Barabási and Pósfai, 2016). The changes observed in these metrics suggest that, while *A. phagocytophilum* infection initially disrupts network structure, making it less cohesive and efficient, the addition of new nodes helps to somewhat mitigate these effects by enhancing connectivity and communication efficiency. This is in agreement with previous reports showing that less robust networks are more susceptible to new associations (Kwon and Cho, 2008; Scheffer et al., 2012; de Morais and Antunes, 2019), making it easier for potential pathogens or commensals to establish and persist within the microbiota. Nevertheless, it is important to acknowledge the limitations inherent to the use of simulated data, such as the inclusion of random nodes. While this approach enables us to explore potential shifts in network dynamics and theoretical resilience, it does not directly replicate biological realities. Consequently, the outcomes should be interpreted as indicative of possible structural responses to hypothetical changes rather than precise predictions of microbial interactions.

The compromised colonization resistance of tick microbiota caused by *A. phagocytophilum* rendering them more susceptible to pathogenic invasion may have implications in the natural environment where tick populations often harbor multiple tick-borne pathogens (TBPs). Co-infection with multiple pathogens is a common phenomenon in ticks (Nieto et al., 2018; Civitello et al., 2010; Moutailler et al., 2016; Hoffmann et al., 2023). It has been suggested that the presence of one pathogen may facilitate the establishment or proliferation of another within the tick microbiota (Gall et al., 2016; Sun et al., 2020). Our results suggest a novel mechanism by which one pathogen infection may decrease the colonization resistance of tick microbiota, favouring subsequent invasion events, that may include TBPs and/or commensals.

Using insights from human microbiota research (Spragge et al., 2023), we can understand potential mechanisms by which compromised colonization resistance in ticks contributes to co-infection dynamics. In humans, diverse microbial communities limit pathogen growth by consuming nutrients that pathogens need, a principle known as nutrient blocking (Spragge et al., 2023). Key species within these communities, especially those closely related to pathogens, are crucial because they increase the overlap in nutrient use, making it harder for pathogens to find the resources they need to grow. Applying this concept to ticks, a compromised colonization resistance - due to reduced robustness and weak community assembly - lessens nutrient blocking, making ticks more susceptible to co-infections. This is because a less diverse microbiome cannot effectively consume all available nutrients, leaving more resources for pathogens. Moreover, the lack of key species that directly compete with pathogens for nutrients may further ease the establishment and spread of these pathogens within the tick.

This may have an impact on disease ecology, as for example, *I. ricinus* and *I. scapularis* ticks are capable of harboring multiple pathogens, thereby increasing the likelihood of co-transmission to humans or animals (Lou et al., 2017). This phenomenon may apply to TBPs other than *A. phagocytophilum*. For example, infection with *B. burgdorferi* has been shown to enhance the transmission of *Babesia microti*, by increasing its basic reproduction number above the threshold for persistence (Diuk-Wasser et al., 2016). Furthermore, co-infection of *B. burgdorferi* and *Rickettsia* spp. in *Ixodes* nymphs results in bacterial replication rates higher than in single infections (Raulf et al., 2018; Sun et al., 2020). Further research could test empirically whether enhanced transmission of *B. microti* is due to a decreased colonization resistance induced by a primary infection with *B. burgdorferi*. While co-infection has been reported to enhance pathogen transmission, it is important to note that pathogens interacting within a population may exhibit complex dynamics, including mutual promotion, competition, or independence (Lou et al., 2017; Sun et al., 2020). Therefore, beyond pairwise interactions, facilitation, and competition summatory effects resulting in reduced colonization resistance may determine specific microbe invasion outcomes.

## 5 Conclusions

Our study revealed that infection with *A. phagocytophilum* alters the microbial community within ticks, potentially affecting their resistance to colonization by other pathogens. While overall microbial diversity remains stable, the composition and network structure of the microbiome showed significant changes post-infection. Our network analysis indicates that *A. phagocytophilum* infection reduces the complexity and robustness of the microbial network, possibly making ticks more susceptible to further pathogenic invasions. These changes suggest a decrease in the microbiotaʼs ability to prevent additional infections, highlighting a potential mechanism for increased co-infection rates in tick populations. This study contributes to understanding how tick-borne diseases might spread more easily due to changes in tick microbiota caused by infection. It suggests that maintaining a healthy and diverse microbial community within ticks could be crucial for controlling the transmission of tick-borne diseases. Further research is necessary to explore the broader implications of these findings across different tick species and pathogens.

## Funding

BIPAR was funded by the French Government’s Investissement d’Avenir program, Laboratoire d’Excellence “Integrative Biology of Emerging Infectious Diseases” (grant no. ANR-10-LABX-62-IBEID). Alejandra Wu-Chuang was supported by the Programa Nacional de Becas de Postgrado en el Exterior “Don Carlos Antonio López” (Grant No. 205/2018). Apolline Maitre is supported by the “Collectivité de Corse”, grant: “Formations superieures” (SGCE-RAPPORT No. 0300).

## Ethical approval

Not applicable.

## CRediT authorship contribution statement

**Lianet Abuin-Denis:** Investigation, Formal analysis, Visualization, Writing – original draft, Writing – review & editing. **Elianne Piloto-Sardiñas:** Formal analysis, Visualization, Writing – original draft, Writing – review & editing. **Apolline Maître:** Visualization, Supervision, Writing – review & editing. **Alejandra Wu-Chuang:** Investigation, Supervision, Formal analysis, Writing – review & editing. **Lourdes Mateos-Hernández:** Investigation, Data curation, Formal analysis, Writing – review & editing. **Dasiel Obregon:** Investigation, Methodology, Writing – review & editing. **Belkis Corona-González:** Supervision, Writing – review & editing. **Andréa Cristina Fogaça:** Supervision, Writing – review & editing. **Vaidas Palinauskas:** Supervision, Methodology, Writing – review & editing. **Justė Aželytė:** Formal analysis, Visualization, Writing – review & editing. **Alina Rodríguez-Mallon:** Supervision, Writing – review & editing. **Alejandro Cabezas-Cruz:** Supervision, Visualization, Resources, Writing – original draft, Writing – review & editing. All authors read and approved the final manuscript.

## Declaration of competing interests

The authors declare that they have no known competing financial interests or personal relationships that could have appeared to influence the work reported in this paper. Given their role as Guest Editor, Alejandro Cabezas-Cruz had no involvement in the peer review of this article and has no access to information regarding its peer review. Full responsibility for the editorial process for this was delegated to Professor Aneta Kostadinova (Editor-in-Chief).

## Data Availability

The data supporting the conclusions of this article are included within the article and its supplementary files. All the datasets shown in the present study can be found at the SRA repository https://www.ncbi.nlm.nih.gov/sra (accession numbers: PRJNA35373 and PRJNA803003).
