# Supplementary material for: Exploring the impact of Anaplasma phagocytophilum on colonization resistance of Ixodes scapularis microbiota using network node manipulation
Source: Curr Res Parasitol Vector Borne Dis. 2024 Apr 28;5:100177. doi: 10.1016/j.crpvbd.2024.100177 (PMC11098721; doi:10.1016/j.crpvbd.2024.100177)
Supplement: Multimedia component 1 [file mmc1.pdf]

Exploring the impact of *Anaplasma phagocytophilum* on colonization resistance of *Ixodes scapularis* microbiota using network node manipulation

Lianet Abuin-Denis [a, b], Elianne Piloto-Sardiñas[b, c], Apolline Maître [b, d, e], Alejandra Wu-Chuang [b], Lourdes Mateos-Hernández [b], Dasiel Obregon [f], Belkis Corona-González [c], Andréa Cristina Fogaça [g], Vaidas Palinauskas [h], Justė Aželytė [h], Alina Rodríguez-Mallon [a], Alejandro Cabezas-Cruz [b*]

Supplementary file S1

R SCRIPT

Betadisper

```
#devtools::install_github("vegandevs/vegan")
library("vegan")
#args(vegan:::plot.betadisper)

data <- read.csv('Table-16S.csv', header = TRUE, row.names = 1)

## Bray-Curtis distances between samples
dis <- vegdist(data[,2:419])

## Calculate multivariate dispersions
mod <- betadisper(dis, data$Condition)
mod

## Perform test
anova(mod)
capture_a <- summary(anova(mod))
capture.output(capture_a, file = "anova results.txt")
## Permutation test for F
permutest(mod, pairwise = TRUE, permutations = 99)
capture_b <- permutest(mod, pairwise = TRUE, permutations = 99)
```

```
capture.output(capture_b, file = "permutation test for F results.txt")
## Tukey's Honest Significant Differences
(mod.HSD <- TukeyHSD(mod))
plot(mod.HSD)
capture_c <- TukeyHSD(mod)
capture.output(capture_c, file = "Tukey's Honest Significant Differences results.txt")
## Plot the groups and distances to centroids on the
## first two PCoA axes
plot(mod)

## with data ellipses instead of hulls

plot(mod, ellipse = TRUE, hull = FALSE, ylim=c(-0.4,0.8)) # 1 sd data ellipse

## Draw a boxplot of the distances to centroid for each group
boxplot(mod)
```

ALDEx2

```
install.packages("BiocManager")
BiocManager::install("ALDEx2")

install.packages("ALDEx2")

library(ALDEx2)
library(ggplot2)
library(tidyverse)
library(matrixStats)
# IMPORT AND FILTER =====
# import ASV/taxa table (sample.id in columns) ====
asv<- read.csv("'Table-16S.csv", header=T, row.names = 1)
asv[1:1,1:4]
dim(asv)
# filter asv with less than 20 reads
```

```
asv2 <- asv[rowSums(asv) >= 10, ]

dim(asv2)

# filter asv appearing in less than 3 samples

asv.f <- asv2[rowCounts(asv2 > 0) >= 2, ]

dim(asv.f)

# import metadata (sample.id in rows, make sure the order by sample.id is the same) ====

metadata <- read.csv("metadata .csv", header=T)

summary(metadata)

# CLR VALUES =====

# It needs a variable with 2 levels to run aldex.clr


conds <- as.character(metadata$twolevels)

a.clr <- aldex.clr(asv, conds, mc.samples=128, denom="all", verbose=F)

clr.values <- aldex.effect(a.clr, include.sample.summary=TRUE)

write.csv(clr.values,"clr-values.csv")

# ALDEX.KW (or t when 2 levels) =====

# run

conds <- as.character(metadata$temp.sex)

aldexo <- aldex(asv.f, conds, mc.samples=128, test="kw", denom="all", verbose=FALSE) # test="t" when 2 levels

# filter output

aldexo.f <- filter(aldexo, kw.ep < 0.05) # can use only one corrected p column name or filter by other values (e.g., 0.10)

aldexo.f[1:4,1:2]; dim(aldexo.f)

max(aldexo.f$kw.ep); max(aldexo.f$kw.eBH) # checking that p values are below chosen threshold (not useful if filtering by both kw and glm)

# add clr values

aldexo.f$asvID <- rownames(aldexo.f) # adding row names as a column

clr.values$asvID <- rownames(clr.values)  # adding row names as a column

aldexo.clr.f <- merge(aldexo.f, clr.values, by=c("asvID"="asvID")) # merging both tables

aldexo.clr.f <- select(aldexo.clr.f, -rab.win.1, -rab.win.2, -rab.all, -diff.btw, -diff.win, -effect, -overlap) # removing unnecessary columns

# -rab.win.treatment1 and 2: edit to actual names
```

colnames(aldexo.clr.f) <- str_remove(colnames(aldexo.clr.f), "rab.sample.") # leaving only sample id as column header

head(aldexo.clr.f)

write.csv(aldexo.clr.f,"aldex-t_XXX.csv")

#Table aldex-kw with all the features

aldexo$asvID <- rownames(aldexo)

aldexo.clr<- merge(aldexo, clr.values, by=c("asvID"="asvID"))

aldexo.clr <- select(aldexo.clr, -rab.win.1, -rab.win.2, -rab.all, -diff.btw, -diff.win, -effect, -overlap)

colnames(aldexo.clr) <- str_remove(colnames(aldexo.clr), "rab.sample.") # leaving only sample id as column header

head(aldexo.clr)

write.csv(aldexo.clr,"aldex-t_XXX_all.csv")

Heatmap

library(Heatplus)

library(gplots)

library(vegan)

library(RColorBrewer)

library(tidyverse)

In this case taxar are columns and samples are rows

data <- read.csv("Table-16S.csv", header = TRUE, row.names = 1)

data[1:3, 1:4]

colnames(data) <- str_remove(colnames(data), "f__") # leaving only sample id as column header

colnames(data) <- str_remove(colnames(data), "g__")

head(data)

CLUSTERING ---

(B) To cluster the SPECIES:

Matrix.dist.g <- vegdist(t(data), method = "euclidean")

col.clus <- hclust(Matrix.dist.g, "aver")

ADD METADATA ---

tempsex.coded <- read.csv("metadata heatmap male.csv", header = TRUE)

```
tempsex.coded
metadata <- list()
metadata$tempsex <- tempsex.coded$temp.sex
metadata$tempsex <- factor(metadata$tempsex)
levels(metadata$tempsex)

# Assign a colour to each level in order
levels(metadata$tempsex)[1] <- "#FFB266"
levels(metadata$tempsex)[2] <- "#FF8000"
levels(metadata$tempsex)[3] <- "#FF0000"
levels(metadata$tempsex)[4] <- "#FFFF66"

# Check that each factor got a colour
levels(metadata$tempsex)
metadata$tempsex <- as.character(metadata$tempsex)

# SCALE COLOR OF HEATMAP
scalecolor <- colorRampPalette(c("blue", "Whitesmoke", "red"), space = "rgb")(100)

# HEATMAP ------------------------------------------------------------------------
windows()
pdf(file = "Heatmap.pdf")
heatmap.2(t(as.matrix(data)),
        Rowv = as.dendrogram(col.clus),
        labRow=as.expression(lapply(colnames(data), function(a) bquote(italic(.(a))))),
        Colv = FALSE,
        na.rm = TRUE,
        col = scalecolor,
        dendrogram = "row",
        trace = "none",
        density.info = "none",
        lhei = c(2, 8),
        key.title = NA,
```

```
        key.xlab = "Center Log Ratio",
        margins = c(0.5, 15),
        ColSideColors = metadata$tempsex,
        main = "Male"
)
dev.off()
```

SPARCC

```
#install.packages ("openxlsx")
#install.packages ("SpiecEasi")
#install.packages ("igraph")

library(devtools)
#install_github("zdk123/SpiecEasi")
library ("openxlsx")
library ("SpiecEasi")
library ("igraph")

latabla <- as.matrix(read.xlsx("Table-16S .xlsx", sheet="Control", startRow = 1, colNames = TRUE,
rowNames = TRUE, detectDates = FALSE, rows = NULL, cols = NULL,
                    check.names = FALSE,
                    namedRegion = NULL, na.strings = "NA", fillMergedCells = FALSE))
# latabla <- latabla[,-ncol(latabla)]
temporal <- rowSums(latabla) ## Removing empty rows
pointer <- which(temporal>0)
latabla3 <- latabla[pointer,] ## Making a new table without the empty rows

latabla <- t(latabla3)

sparcc.latabla <- sparcc(latabla, iter=20, inner_iter=10, th=0.3)
sparcc.graph <- sparcc.latabla$Cor
```

```
## correlation cutoff of 0.5

sparcc.cutoff <- 0.5


## Obtain positive and negative interactions

sparcc.graph <- ifelse(abs(sparcc.latabla$Cor) >= sparcc.cutoff, sparcc.latabla$Cor, 0)


## Or use this for only positive interactions

#sparcc.graph[sparcc.graph<0.50] <- 0


colnames(sparcc.graph) <- colnames(latabla)

rownames(sparcc.graph) <- colnames(latabla)

diag(sparcc.graph) <- 0


# Create igraph objects and export to "graphml" for Gephi

ig.sparcc <- graph.adjacency(sparcc.graph, mode = "undirected", weighted = TRUE, diag = FALSE,
add.colnames = TRUE)

write_graph(ig.sparcc, "Control.graphml", format = c("graphml"))
```

NETCOMI

```
install.packages("devtools")


devtools::install_github("stefpeschel/NetCoMi", dependencies = TRUE,

                    # repos = c("https://cloud.r-project.org/",

                             BiocManager::repositories()))


library(SpiecEasi)

library(NetCoMi)

library ("openxlsx")


HM <- as.matrix(read.xlsx("Table-16S.xlsx", sheet="Uninfected", startRow = 1, colNames = TRUE,
rowNames = TRUE, detectDates = FALSE, rows = NULL, cols = NULL,

                        check.names = FALSE,

                        namedRegion = NULL, na.strings = "NA", fillMergedCells = FALSE))
```

```
RB <- as.matrix(read.xlsx("Table-16S.xlsx", sheet="Infected", startRow = 1, colNames = TRUE,
rowNames = TRUE, detectDates = FALSE, rows = NULL, cols = NULL,
                    check.names = FALSE,
                    namedRegion = NULL, na.strings = "NA", fillMergedCells = FALSE))

devtools::install_github("vmikk/metagMisc")
library(metagMisc)

net_season <- netConstruct(data = Uninfected,
                    data2 = Infected,
                    measure = "sparcc",
                    normMethod = "none",
                    zeroMethod = "none",
                    sparsMethod = "threshold",
                    thresh = 0.50,
                    dissFunc = "signed",
                    verbose = 3,
                    seed = 123456)

props_season <- netAnalyze(net_season,
                    centrLCC = FALSE,
                    avDissIgnoreInf = FALSE,
                    sPathNorm = FALSE,
                    clustMethod = "cluster_fast_greedy",
                    hubPar = c("eigenvector"),
                    hubQuant = 0.50,
                    lnormFit = FALSE,
                    normDeg = FALSE,
                    normBetw = FALSE,
                    normClose = FALSE,
                    normEigen = FALSE)

summary(props_season)
```

```
comp_season <- netCompare(props_season, permTest = FALSE, verbose = FALSE)

comp_seasonsummary <- summary(comp_season,
        groupNames = c("Uninfected", "Infected"),
        showCentr = c("degree", "between", "closeness"),
        numbNodes = 5)

summary(comp_season, pAdjust = TRUE,
        groupNames = c("Uninfected", "Infected"), digitsPval = 6)

net_season_pears <- netConstruct(data = Uninfected,
                                 data2 = Infected,
                                 measure = "pearson",
                                 normMethod = "mclr",
                                 sparsMethod = "none",
                                 thresh = 0.50,
                                 verbose = 3)

diff_season <- diffnet(net_season_pears,
                       diffMethod = "fisherTest",
                       adjust = "lfdr")

pdf(file = "diff network .pdf",
    width = 6,
    height = 4)

x11()
plot(diff_season,
     cexNodes = 0.8,
     cexLegend = 1,
     cexTitle = 1,
     mar = c(2,2,8,5),
     legendGroupnames = c("Uninfected", "Infected"),
```

```
    legendPos = c(0.4,1))
props_season_pears <- netAnalyze(net_season_pears,
                         clustMethod = "cluster_fast_greedy",
                         weightDeg = TRUE,
                         normDeg = FALSE)
dev.off()

capture.output(summary(comp_season,
                  groupNames = c("Uninfected", "Infected"),
                  showCentr = c("degree", "between", "closeness"),
                  numbNodes = 5),file="comparison Uninfected-Infected")
save.image("jaccard-corse-Uninfected-Infected.RData")
```

Node Addition

```
# Load the igraph package
library(igraph)
library(ggplot2)
library(cowplot)
library(coin) # For the permutation test
library(boot) # For bootstrapping
library(stats) # For p.adjust()

# Load the edge list file
edge_list <- read.table("Table-16S.csv", header = TRUE, sep = ",")

# Convert node names to character strings
edge_list$Source <- as.character(edge_list$Source)
edge_list$Target <- as.character(edge_list$Target)

# Create a vector of all the vertex names
all.vertices <- unique(c(edge_list$Source, edge_list$Target))
```

```
# Create the graph object with all vertices
g <- graph_from_data_frame(edge_list, directed = FALSE, vertices = all.vertices)

# Calculate the network's robustness to node addition
n.sim <- 10 # Number of simulations
n.add <- 50 # Number of nodes to add in each simulation
robustness <- numeric(n.add*n.sim)
path.lengths <- numeric(n.add*n.sim)
new.nodes <- paste0("NewNode", 1:n.add)

for (i in 1:n.add) {
  for (j in 1:n.sim) {
    # Add the new nodes and connect them to the existing network
    g.new <- add.vertices(g, i, name = new.nodes[1:i])
    new.edges <- cbind(sample(new.nodes[1:i], i, replace = TRUE), sample(all.vertices, i, replace =
TRUE))
    g.new <- add_edges(g.new, new.edges)

    # Calculate the size of the largest connected component
    ccs <- clusters(g.new)
    max.cc <- max(ccs$csize)
    robustness[(i-1)*n.sim+j] <- max.cc

    # Calculate the average path length
    path.lengths[(i-1)*n.sim+j] <- average.path.length(g.new)
  }
}

# Perform one-sample Wilcoxon signed-rank tests and extract p-values
robustness.wilcox <- wilcox.test(robustness, mu = 0, exact = FALSE)
path.lengths.wilcox <- wilcox.test(path.lengths, mu = 0, exact = FALSE)
robustness.pvalue <- robustness.wilcox$p.value
path.lengths.pvalue <- path.lengths.wilcox$p.value
```

```
# Calculate BH adjusted p-values
pvalues <- c(robustness.pvalue, path.lengths.pvalue)
adjusted_pvalues <- p.adjust(pvalues, method = "BH")
robustness.adjusted_pvalue <- adjusted_pvalues[1]
path.lengths.adjusted_pvalue <- adjusted_pvalues[2]

# Perform bootstrapping for confidence intervals
boot_func <- function(data, indices) {
  return(mean(data[indices]))
}
robustness.boot <- boot(robustness, boot_func, R = 1000)
path.lengths.boot <- boot(path.lengths, boot_func, R = 1000)

# Create data frames for ggplot
robustness.df <- data.frame(nodes_added = rep(1:n.add, each=n.sim), robustness = robustness)
path.lengths.df <- data.frame(nodes_added = rep(1:n.add, each=n.sim), path_lengths = path.lengths)

# Fit the linear model and extract the coefficients
path_lengths.lm <- lm(path_lengths ~ nodes_added, data = path.lengths.df)
path_lengths_R2 <- summary(path_lengths.lm)$r.squared
path_lengths_coef <- coef(path_lengths.lm)[2]
path_lengths_pvalue <- coef(summary(path_lengths.lm))[2, "Pr(>|t|)"]

robustness.lm <- lm(robustness ~ nodes_added, data = robustness.df)
robustness_R2 <- summary(robustness.lm)$r.squared
robustness_coef <- coef(robustness.lm)[2]
robustness_pvalue <- coef(summary(robustness.lm))[2, "Pr(>|t|)"]

# Customize the ggplot objects for publication
robustness.plot <- ggplot(robustness.df, aes(x = nodes_added, y = robustness)) +
  geom_point(alpha = 0.5, size = 1) +
  stat_smooth(method = "lm", se = TRUE, col = "#1B9E77") +
```

```
  labs(x = "Nodes Added", y = "LCC Size") +
  theme_classic(base_size = 10) +
  theme(panel.grid = element_blank()) +
  theme(axis.line = element_line(colour = "black", size = 0.5)) +
  scale_x_continuous(expand = c(0, 1), breaks = seq(0, 50, by = 5)) +
  geom_text(aes(x = 1, y = max(robustness), label = paste0("R2 : ", format(robustness_R2, digits = 3), "\n", "p-value : ", format(robustness_pvalue, digits = 3), "\n", "Coef : ", format(coef(robustness.lm)[2], digits = 3))), hjust = 0, vjust = 1, size = 3) +
  geom_text(aes(x = 15, y = max(robustness), label = paste0("p-adj(BH): ", format(robustness.adjusted_pvalue, digits = 3))), hjust = 0, vjust = 1, size = 3)

path.lengths.plot <- ggplot(path.lengths.df, aes(x = nodes_added, y = path_lengths)) +
  geom_point(alpha = 0.5, size = 1) +
  stat_smooth(method = "lm", se = TRUE, col = "#D95F02") +
  labs(x = "Nodes Added", y = "Avg. Path Length") +
  theme_classic(base_size = 10) +
  theme(panel.grid = element_blank()) +
  theme(axis.line = element_line(colour = "black", size = 0.5)) +
  scale_x_continuous(expand = c(0, 1), breaks = seq(0, 50, by = 5)) +
  geom_text(aes(x = 1, y = max(path_lengths), label = paste0("R2 : ", format(path_lengths_R2, digits = 3), "\n", "p-value : ", format(path_lengths_pvalue, digits = 3), "\n", "Coef : ", format(coef(path_lengths.lm)[2], digits = 3))), hjust = 0, vjust = 1, size = 3) +
  geom_text(aes(x = 15, y = max(path_lengths), label = paste0("p-adj(BH): ", format(path.lengths.adjusted_pvalue, digits = 3))), hjust = 0, vjust = 1, size = 3)

# Combine the plots
combined_plot <- plot_grid(robustness.plot, path.lengths.plot, ncol = 1, align = "v", rel_heights = c(1, 1))

# Show the plot in RStudio console
print(combined_plot)

# Write R-squared and p-value to txt file
write(paste0("Robustness R-squared: ", robustness_R2, "; p-value: ", robustness_pvalue, "\n"), file = "UNF-LM-NodeAddition.txt")
```

```
write(paste0("Path lengths R-squared: ", path_lengths_R2, "; p-value: ", path_lengths_pvalue, "\n"),
file = "UNF-LM-NodeAddition.txt", append = TRUE)


# Save the plot as a high-resolution image

ggsave("UNF-2.Node-Addition_plots.pdf", combined_plot, width = 4, height = 6, dpi = 300)
```

NODE REMOVAL

```
# Load packages and data

library(igraph)

library(NetSwan)

library(data.table)

library(MASS)


elec<- read.csv("Table-16S.csv", header=T)

elec<-as.matrix(elec)

gra<-graph.edgelist(elec, directed=FALSE)


# Calculate connectivity loss

f4<-swan_combinatory(gra,10)

f4<-as.data.frame(f4)

setnames(f4, old = c('V1','V2','V3','V4','V5'), new = c('fraction_nodes','loss_connec_BNC

','loss_connec_DEG','loss_connec_Cascading','loss_connec_Random'))

write.table(f4, file="f4.csv", sep=",", row.names=FALSE, col.names=TRUE)


# Calculate standard errors

se <- apply(f4[, -1], 2, function(x) qnorm(0.975) * sd(x) / sqrt(nrow(f4)))


# Plot the data with error bars

par(family = "sans")

par(cex.lab = 1, cex.axis = 1, cex.main = 1)

plot(f4[,1], f4[,5], type='l', lwd=2, col='#1f77b4', xlab="Fraction of nodes removed",

    ylab="Connectivity loss", main="UNF network robustness")

lines(f4[,1], f4[,3], type='l', lwd=2, col='#ff7f0e')
```

```
lines(f4[,1], f4[,4], type='l', lwd=2, col='#2ca02c')
lines(f4[,1], f4[,2], type='l', lwd=2, col='#d62728')

# Add shaded confidence intervals
polygon(c(f4[,1], rev(f4[,1])),
        c(f4[,5] + se[1], rev(f4[,5] - se[1])),
        col = adjustcolor('#1f77b4', alpha.f = 0.2),
        border = NA)
polygon(c(f4[,1], rev(f4[,1])),
        c(f4[,3] + se[2], rev(f4[,3] - se[2])),
        col = adjustcolor('#ff7f0e', alpha.f = 0.2),
        border = NA)
polygon(c(f4[,1], rev(f4[,1])),
        c(f4[,4] + se[3], rev(f4[,4] - se[3])),
        col = adjustcolor('#2ca02c', alpha.f = 0.2),
        border = NA)
polygon(c(f4[,1], rev(f4[,1])),
        c(f4[,2] + se[4], rev(f4[,2] - se[4])),
        col = adjustcolor('#d62728', alpha.f = 0.2),
        border = NA)

# Add legend
legend('bottomright',c("Random", "Betweenness", "Degree", "Cascading"),
       lty=c(1,1,1,1), pch=c(1,1,1,1), cex=0.8,
       col=c("#1f77b4","#d62728","#ff7f0e", "#2ca02c"))
```
